# Multidisciplinary Approach for Dental Management of Congenital Insensitivity to Pain with Anhidrosis: Clinical Case Report with 12-Month Follow-Up

**DOI:** 10.3390/dj14010068

**Published:** 2026-01-20

**Authors:** Almoataz B. A. T. Abdel-bari, Mohamed Fawzy, Khaled A. Saad, Hatem A. Alhadainy

**Affiliations:** 1 Department of Oral and Maxillofacial Surgery, Faculty of Dentistry, Tanta University, Tanta 31527, Egypt; moataz_abdelbari@dent.tanta.edu.eg (A.B.A.T.A.-b.); khaled.elazab@dent.tanta.edu.eg (K.A.S.); 2 Department of Endodontics, Faculty of Dentistry, Tanta University, Tanta 31527, Egypt; mohamed.ali.mansour@dent.tanta.edu.eg; 3 Division of Endodontics, Department of Dentistry, University of North Carolina, Chapel Hill, NC 27514, USA

**Keywords:** congenital analgesia, congenital insensitivity to pain with anhidrosis (CIPA), hereditary sensory autonomic neuropathy, intraoral scanner, wearable temperature monitor

## Abstract

**Background:** Congenital Insensitivity to Pain and Anhidrosis (CIPA) is a rare autosomal recessive disorder characterized by congenital analgesia, anhidrosis, and multisystem involvement affecting the musculoskeletal, cutaneous, oral, and para-oral structures. This case report describes the oral phenotype and multidisciplinary clinical management of a child with CIPA. **Case Description:** A 9-year-old boy presented with poor oral hygiene, multiple severely damaged teeth, masticatory difficulty, limited mouth opening, impaired bolus control, and para-oral traumatic injuries. Medical and orthopedic history indicated recurrent painless fractures, self-inflicted injuries, cutaneous scarring, and recurrent hyperpyrexia. Oral self-injury associated with CIPA was suspected and supported by the Nociception Assessment Test and Minor’s Iodine–Starch Test. Although the clinical findings were suggestive of CIPA, the diagnosis remained presumptive due to the absence of confirmatory molecular or histopathological testing. **Management:** A wearable wireless continuous temperature-monitoring device was prescribed to assist in tracking hyperpyrexia associated with CIPA (RHA-CIPA). A conservative, staged, multidisciplinary treatment was planned rather than full-mouth extraction, emphasizing prevention of dental sepsis and mitigation of future self-injury. Dental procedures were performed under local anesthesia to manage discomfort related to tactile hyperesthesia. To reduce nocturnal biting and oral trauma, a hard acrylic occlusal protector was fabricated using an intraoral scanner and a 3D-printed cast. The patient was followed for 12 months. **Outcomes:** At the 12-month follow-up, clinical improvement was observed, with particularly notable gains in cheek elasticity and soft tissue resilience. **Conclusions:** This case highlights the considerable challenges involved in the interdisciplinary management of children with CIPA, including oral self-injury prevention, limited mouth opening, and the necessity of close coordination with medical specialties. These findings are descriptive observations of a single case and do not establish efficacy or generalizability of any intervention.

## 1. Introduction

Congenital Insensitivity to Pain and Anhidrosis (CIPA) is a rare, autosomal recessive neurodevelopmental disorder distinguished by a profound absence of nociception and markedly impaired sudomotor function. These deficits confer a substantially increased susceptibility to accidental injuries, repetitive self-inflicted trauma, thermal burns, and progressive orthopedic complications, including recurrent fractures and joint degeneration. Earlier epidemiological observations indicated that mortality among affected infants and young children approached 20% before the age of 3 years; however, more recent clinical experience suggests that outcomes have improved considerably due to advances in early diagnosis, vigilant clinical surveillance, and multidisciplinary supportive care [1,2]. Defective thermoregulation frequently manifests as recurrent episodes of hyperpyrexia, events that can escalate rapidly to a fatal risk if unrecognized or inadequately managed. Rigorous and continuous monitoring of recurrent hyperpyrexia associated with CIPA (RHA-CIPA) is essential for mitigating morbidity and minimizing the risk of fatal complications in this population.

CIPA remains a rare clinical entity, with an estimated global prevalence of approximately 1 per 125 million individuals and fewer than 300 rigorously documented cases reported in the medical literature to date [3,4]. The disorder is most commonly attributable to loss-of-function mutations in the Neurotrophic Tyrosine Kinase Receptor type 1 (NTRK1) gene, the receptor essential for nerve growth factor signaling. Disruption of this pathway compromises the development, maintenance, and survival of nociceptive neurons, eccrine sweat glands, and broader components of the autonomic nervous system, thereby producing the characteristic constellation of analgesia and anhidrosis [5]. CIPA is classified as type four in a group of disorders of Hereditary Sensory and Autonomic Neuropathy (HSAN IV). Congenital insensitivity to pain without anhidrosis encompasses a heterogeneous condition in which nociceptive signaling is selectively impaired while sudomotor function remains intact. Consequently, the presence of anhidrosis represents a critical diagnostic criterion distinguishing CIPA from other phenotypic variants of congenital pain insensitivity [6].

Oral and para-oral tissues are vulnerable to trauma in CIPA patients due to absent nociception and behavioral self-injury. Reported injuries include tongue and lip ulcerations, cheek biting, dental fractures, as well as the ears, eyes, and nose. Painless jaw fractures, osteomyelitis, and wound sepsis are common with this disorder [7]. To control self-trauma and oral sepsis, dental practitioners need to be acquainted with CIPA and its special management requirements.

Oral and para-oral structures are particularly susceptible to injury in individuals with CIPA, owing to the profound absence of nociceptive feedback and the frequent occurrence of repetitive self-injurious behaviors. Documented manifestations include chronic ulcerations of the tongue and lips, habitual cheek biting, coronal and root fractures, and traumatic lesions involving adjacent craniofacial sites such as the external ear, ocular adnexa, and nasal tissues. More severe complications, most notably painless mandibular or maxillary fractures, secondary osteomyelitis, and recurrent wound sepsis, are well-recognized clinical sequelae in this population [7]. Consequently, effective prevention and management of self-inflicted oral trauma and associated infectious morbidity require dental practitioners to possess a thorough understanding of CIPA’s pathophysiology and its distinct clinical management considerations.

The existing dental literature concerning CIPA (HSAN IV) remains sparse, and current knowledge regarding evidence-based dental management in this population is notably limited. Therefore, we present a comprehensive case description and a structured dental management strategy for a pediatric patient diagnosed with CIPA. The patient was monitored longitudinally over 12 months, underscoring the critical role of dental practitioners in the early recognition, ongoing surveillance, and multidisciplinary management of this neurodevelopmental disorder.

## 2. Case Description

### 2.1. Chief Complaint

A 9-year-old male patient presented to the outpatient clinic of the Department of Oral and Maxillofacial Surgery, accompanied by his parents. The primary concern was the need for full-mouth dental clearance in preparation for the construction of a complete denture. The parents reported that the child’s oral health issues were affecting his overall well-being. Specifically, the child exhibited multiple carious and missing teeth, difficulty with chewing, limited mouth opening, an inability to retain food within the oral cavity, and poor oral hygiene.

### 2.2. Medical History

The patient’s parents declared that they are first cousins. They noted that the patient did not react to painful stimuli during routine activities such as playing, falling, or interacting with his environment. Additionally, the child had experienced recurrent bone fractures, which were attributed to the lack of pain perception, as well as multiple episodes of disturbance of body temperature occurring at least once per month, without an identifiable cause.

**Figure 1:**
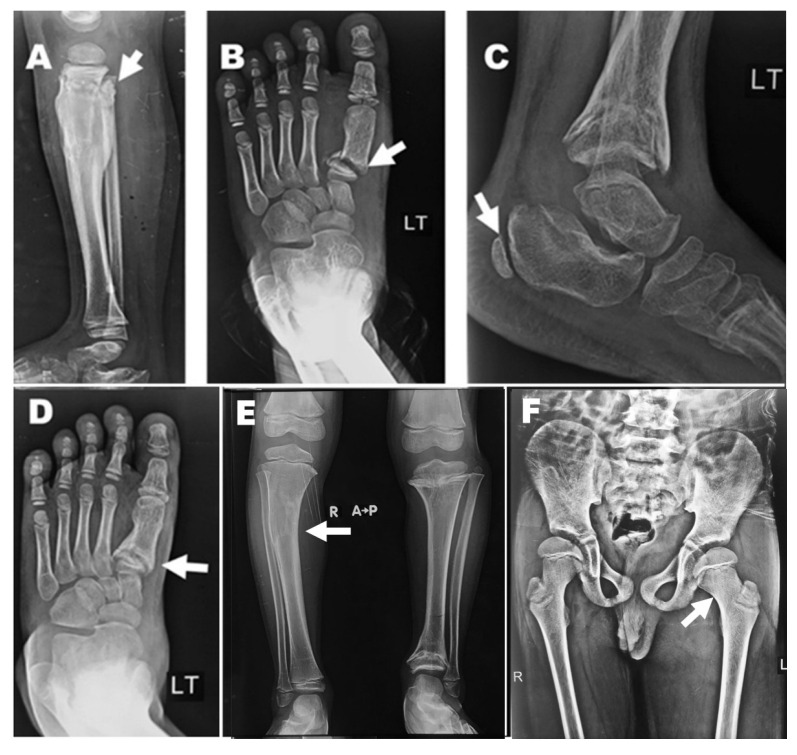
Orthopedic radiographs, the arrows show hypertrophic bone callus formation (**A**) left 1st metatarsal fracture (**B**), left calcaneal fracture (**C**), imperfect bone consolidation of 1st metatarsal fracture (**D**), right bowed legs with shorter left leg (**E**), deformity of the left hip where there is an increased angle between the femoral neck and femoral shaft (**F**).

The patient’s orthopedic history indicated notable multiple fractures of the right tibia. The fractures were surgically treated at the age of 4 years with elastic intramedullary nailing. However, the tibia was refractured two years later and was managed conservatively through immobilization, with subsequent formation of hypertrophic bone callus following healing. Radiographic imaging of the affected limb revealed the presence of hypertrophic bone calluses, as well as a deformity of the left hip, characterized by an increased angle between the femoral neck and femoral shaft (Figure 1).

**Figure 2:**
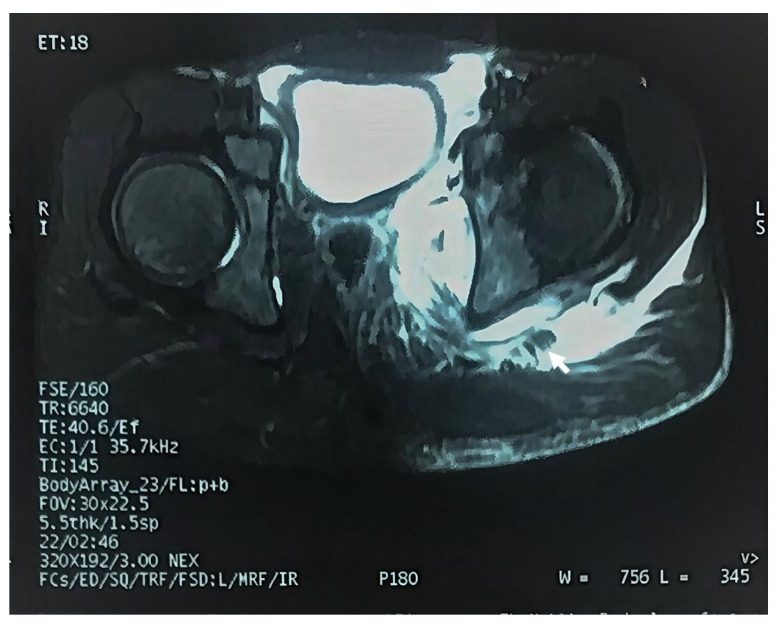
A contrast-enhanced MRI of the hip joint showing deep left pelvic inflammatory process (Arrow).

Left hip joint contrast-enhanced Magnetic Resonance Imaging (MRI) identified a localized, lobulated fluid signal within the inner aspect of the left acetabulum, along with a mild pelvic fluid collection, suggestive of a deep pelvic inflammatory process (Figure 2). The multiple bone fractures resulted in significant physical disability, compounded by cutaneous injuries of unclear etiology.

### 2.3. General Examination

**Figure 3:**
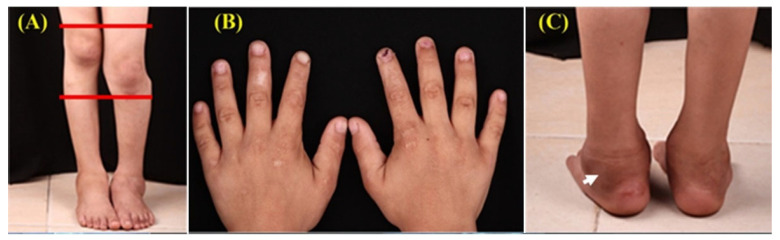
Clinical photographs show asymmetrical legs with length discrepancy (**A**), finger atrophic scars in both hands (**B**), and left lower limb edema (**C**) (Arrow).

The child was hyperactive, mildly intellectually delayed, and of appropriate height and weight. Examination revealed asymmetric lower limbs, a limping gait, atrophic scars on hands and feet, and left-leg edema (Figure 3).

### 2.4. Oral Examination

**Figure 4:**
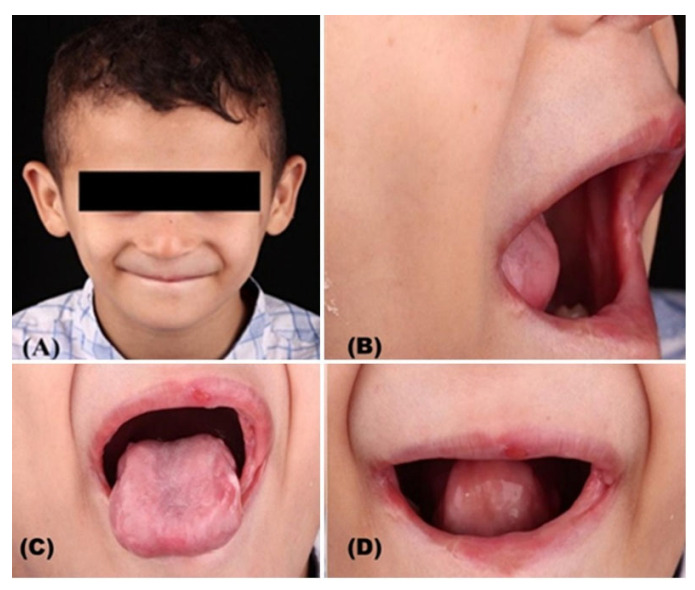
Clinical oral examination: Extra-oral frontal view of the child (**A**), marked fibrous band of left buccal mucosa (**B**), atrophic, ulcerated, and depapillated tongue (**C**), close-up view of both lips showing catastrophic scarring of the lower lip (**D**).

Intraoral examination revealed bilateral buccal mucosal fibrosis and de-papillated ulcerated tongue, indicating self-inflicted injuries likely due to pain insensitivity. The tongue was touched with a sharp dental instrument and did not provoke any bodily or emotional reaction. Extraoral findings included extensive scarring of the lower lip (Figure 4). Despite normal temporomandibular joint findings, digital palpation revealed non-tender masticatory muscles, a tense masseter muscle, and strong fibrous bands of the cheek, which limited the mouth opening, preventing the acquisition of high-quality intraoral photographs.

Orthopantomography showed severe decay in the first permanent molars and retained root fragments. The patient had intentionally extracted his primary lower canines and lower first molars at approximately 7 years of age, resulting in a flattened lower alveolar ridge in those regions.

### 2.5. Diagnostic Assessment

A differential diagnosis included subtypes of Hereditary Sensory and Autonomic Neuropathy (HSAN), forms of congenital insensitivity to pain without anhidrosis, severe neuropathies, acquired sensory neuropathies, neurodevelopmental disorders associated with self-injurious behavior, and non-accidental injury. These differential diagnoses were excluded, and a diagnosis of oral self-injury secondary to CIPA was considered based on a comprehensive review of the patient’s medical history, characteristic clinical findings, and orthopedic evaluations. Two diagnostic assessments were used to support this diagnosis. The Nociception Assessment Test was conducted by applying a pressure of 5–7 kg for several seconds to the nail bed using a pen; the patient showed neither withdrawal nor pain response, supporting the absence of nociception. Anhidrosis was evaluated using Minor’s Iodine–Starch Test, in which the skin was coated with iodine solution, allowed to dry, and then dusted with starch powder. After exposure to controlled heat, no dark blue or black discoloration developed, indicating a lack of sweat production.

The definitive diagnosis of CIPA requires molecular confirmation through identification of pathogenic variants in the NTRK1 gene or histopathological assessment of intraepidermal nerve fiber density and autonomic innervation via skin biopsy. In the current case, parental refusal prevented the pursuit of genetic testing, and no histopathological evaluation was conducted; consequently, the diagnosis remains presumptive in the absence of confirmatory investigations.

Guided by the clinical findings supporting this presumptive diagnosis, we elected not to proceed with full-mouth extraction and complete denture fabrication as initially requested by the parents. Instead, a conservative management plan was adopted, prioritizing the prevention of dental sepsis and mitigation of future self-injurious behavior. The clinical presentation, diagnostic considerations, and proposed treatment strategy were thoroughly explained to the patient’s parents, and written informed consent was obtained for all dental procedures, the use of the wearable temperature-monitoring device, and the inclusion of clinical information and photographs in this report.

## 3. Dental Management

### 3.1. Temperature Monitoring

To help manage RHA-CIPA, a wearable continuous temperature-monitoring device (Celsium^®^, SMARTR Health Ltd., London, UK) was prescribed. This device is a waterproof underarm sensor that wirelessly transmits temperature data to a smartphone via Bluetooth, enabling real-time physiological monitoring. The parents set up the device, and all readings remained accessible to the parents via the mobile application and were shared with the patient’s pediatrician.

### 3.2. Treatment Plan

We planned a treatment approach through multiple short chair-time sessions using stress relief methods, “Tell–Show–Do,” and positive reinforcement was employed for all visits to reduce anxiety. We scheduled multiple short visits (20–30 min), used brief working intervals, employed gauze rolls and gentle finger retraction, and, when tolerated, pediatric mouth props to manage limited mouth opening. Procedures were performed under continuous clinical observation with parental presence, and local anesthesia was administered if tactile hyperesthesia or defensive movements were elicited. The following is the multidisciplinary timeline of dental management, including surgical, endodontic, restorative, and orthodontic treatments.

### 3.3. Visit 1: Surgical Session

Extraction of the chronically infected remaining root of the lower left first molar was attempted without local anesthesia. The patient expressed unpleasant feelings, which necessitated the administration of a field-block anesthesia of 2% lidocaine and 1:100,000 epinephrine (Lidocaine, Alex Pharma, Alexandria, Egypt). After extraction, the socket was sutured with resorbable sutures, and a small pressure pack was applied over the wound. Amoxicillin 40 mg/kg/day, divided into 3 doses, was prescribed for one day before extraction and continued for 5 days postoperatively to control sepsis risk.

### 3.4. Visit 2: Endodontic Session

The patient was subsequently referred to the Department of Endodontics for treatment of the maxillary left first molar. Limited mouth opening compromised patient cooperation and posed challenges to ensuring patient safety, performing clinical procedures, and maintaining adequate intraoral access. Consequently, high-quality intraoral photographs could not be obtained. The clinical procedures were completed using controlled retraction and brief working intervals.

Endodontic treatment was initially initiated without the use of local anesthesia; however, when the endodontic file approached the periapical region, the patient produced a brief chuckling response, indicating hyperesthesia to tactile or pressure stimuli. Consequently, an intra-pulpal injection of 2% lidocaine with 1:100,000 epinephrine (Lidocaine, Alex Pharma, Alexandria, Egypt) was administered to ensure adequate anesthesia and to prevent involuntary movements during instrumentation. Working length determination was performed using an electronic apex locator (Root ZX, J. Morita Corp., Osaka, Japan), establishing the length at 1 mm short of the radiographic apex. Chemo-mechanical preparation of the canal was completed using the ProTaper Universal system (Dentsply, Tulsa, OK, USA) to an F4 file size, with continuous irrigation using 2.5% sodium hypochlorite throughout instrumentation. Final irrigation was performed with 17% ethylenediaminetetraacetic acid (EDTA) (META Biomed Co., Colmar, PA, USA) to remove the smear layer. Apical patency was maintained with a size #10 K-file, after which the canal was flushed with normal saline and dried with paper points. Obturation was performed using gutta-percha and AH-26 sealer (Dentsply, DeTrey GmbH, Tulsa, OK, USA) via the lateral condensation technique. The coronal access was subsequently sealed with a glass ionomer restorative material (Prima Dental, Gloucester, GL2 2HA, UK) to ensure an immediate coronal seal. The patient’s limited mouth opening caused clinical challenges; this constraint was particularly evident during endodontic treatment of the maxillary molars, where access and visibility were substantially compromised.

### 3.5. Visit 3: Restorative Session

Glass ionomer restorative material was used for the restoration of carious lesions. Smoothing of sharp dental surfaces and placement of restorations were performed without local anesthesia, as the patient exhibited no pain-related responses during these procedures. However, the patient’s markedly restricted mouth opening posed clinical challenges for obtaining high-quality intraoral photographs and complicating routine operative procedures.

### 3.6. Visit 4: Orthodontic/Behavioral Management Session

The patient was then referred to the Department of Orthodontics, where a therapeutic program was advocated to consist of patient and family member counselling as well as masticatory muscle-stretching exercises. Counseling was targeted at altering maladaptive habits and behaviors, employing techniques such as habit reversal and habit termination. To stretch the masticatory muscles, the patient was instructed to open his mouth by placing his thumbs on the upper incisive area and his index fingers on the lower incisive area. The exercises were performed under parental supervision for 3–4 times daily. Each stretch was held for 15–30 s, followed by a period of relaxation between repetitions.

### 3.7. Occlusal Protector Fabrication

To control the nocturnal biting/parasomnia of the cheek, lip, and tongue, a hard occlusal acrylic protector was planned. Due to the oral thick fibrous band, it was not feasible to place an impression tray in the patient’s mouth to take a conventional impression, despite repeated tray modification. Accordingly, we used an intra-oral scanner (TRIOS 3Shape, Dandy, Paris, France) to take a digital impression, followed by the construction of a 3D-printed cast.

**Figure 5:**
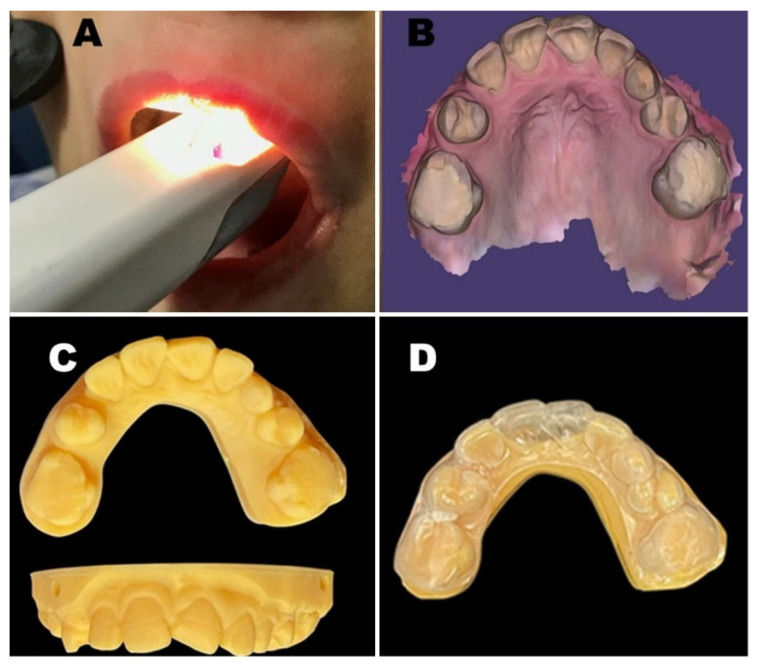
Management of the patient: The Trios 3Shape Scanner during the scanning (**A**), Virtual 3-D Model of digital impression showing elimination of any sharp edges of the teeth before acrylic protector manufacturing (**B**), 3D-printed cast for upper arch (**C**), upper hard acrylic protector (**D**).

A cold-cure hard acrylic occlusal guard of 3 mm thickness was constructed to resist nocturnal biting and limit oral self-injury. It was designed without palatal coverage for comfort and speech, with superior retention in the maxillary arch to allow for erupting teeth. The appliance was highly polished to reduce irritation and plaque buildup. The patient was instructed to wear the occlusal guard at night, and the mother was advised to rinse it after each use by brushing with soap, store it in a dry and ventilated area, and perform weekly cleansing with a non-abrasive solution (Figure 5). A new acrylic guard will ultimately be essential to accommodate the developing teeth. For the patient’s safety, strict oral hygiene measures, meticulous dental monitoring, and consistent use of the occlusal guard were advised to the child’s mother.

### 3.8. Follow up

The patient underwent scheduled follow-up recalls at 3-, 6-, and 12 months post-treatment. We did not participate in the generation, maintenance, or verification of the device-recorded temperature logs, alarm-activation frequencies, or continuous temperature time-series data that were made solely available to the patient’s parents. Consequently, all assessments regarding the device’s performance characteristics or its potential clinical utility relied exclusively on reports of the patient’s parents and should therefore be regarded as subjective observations rather than empirically substantiated evidence.

## 4. Outcomes

**Figure 6:**
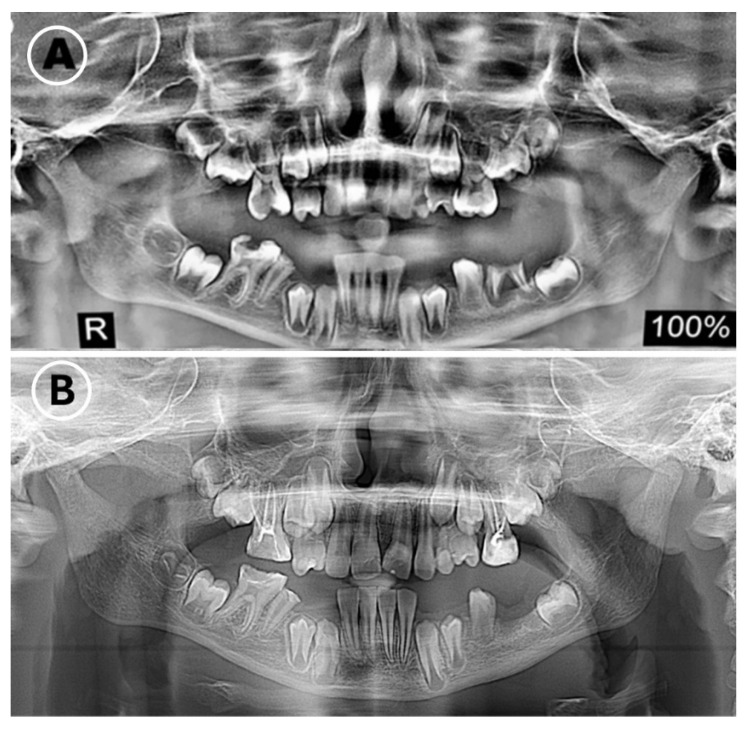
Pre-and post-operative panoramic radiographs showing multiple carious teeth and chronically infected lower left first molar remaining roots (**A**), and the treatment performed on all affected teeth (**B**).

Figure 6 shows full mouth panoramic radiographs illustrating dental conditions before and after treatment.

### 4.1. The 3-Month Recall Visit

The patient reported no discomfort following restorative procedures, and clinical evaluation demonstrated improved oral health status with normal healing of the extraction sites. According to the patient’s mother, the child consistently performed the prescribed masticatory muscle-stretching exercises. These observations underscore that behavioral modifications do not occur spontaneously; rather, they require sustained patient cooperation supported by active parental involvement to achieve meaningful change. The patient’s parents also reported that the wearable temperature-monitoring device was beneficial in tracking temperature fluctuations, thereby facilitating timely recognition of episodes suggestive of thermoregulatory instability. However, parents’ reports concerning the performance or clinical utility of this device are based solely on parental observations and should be interpreted as anecdotal rather than empirically validated evidence.

### 4.2. The 6-Month Recall Visit

The patient demonstrated consistent and favorable tolerance of the hard acrylic occlusal guard throughout the 6-month follow-up period. Clinical assessment revealed a substantial reduction in both lip and tongue biting, with complete resolution of the previously documented tongue ulcer and associated soft-tissue trauma. These outcomes are attributable to the mechanical barrier provided by the occlusal guard, which effectively limited nocturnal parafunctional activity and mitigated self-injurious contact between the dentition and oral soft tissues. Parental reports, corroborated by clinical observation, confirmed that the patient adhered to the recommended nightly wear protocol, contributing to the sustained therapeutic benefit.

### 4.3. The 12-Month Recall Visit

**Figure 7:**
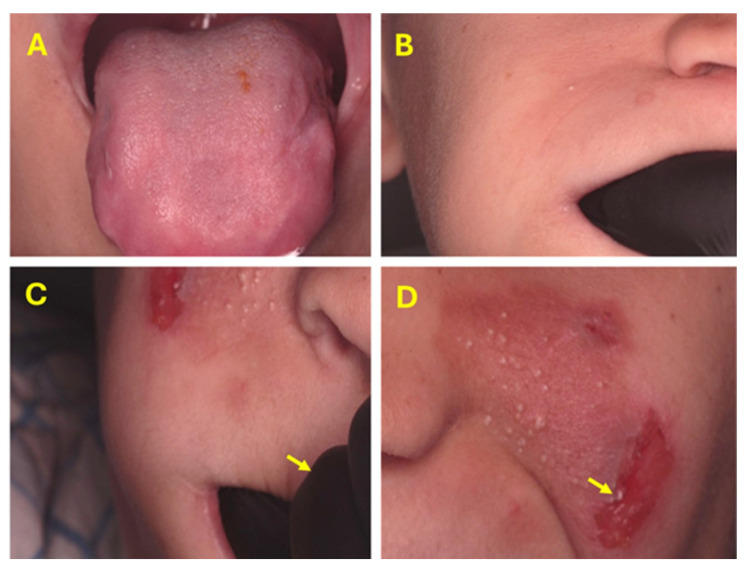
Clinical photographs for 12-month follow-up show healing of tongue ulcers (**A**). Cheek elasticity improvement, indicating progressive tissue resilience (**B**) as displayed by lateral retraction with the operator’s finger (black glove), (**C**). A new facial self-inflicted ulcer is also evident (arrow) (**D**).

At the 12-month follow-up, the patient demonstrated sustained control of lip and tongue biting, complete resolution of previously documented oral ulcers, and full healing of the extraction site. A particularly notable clinical finding was the marked improvement in cheek elasticity and soft-tissue resilience (Figure 7). Although preoperative quantitative measurements of maximum mouth opening were not available for direct comparison, qualitative assessment revealed enhanced cheek compliance. This was evaluated by placing the operator’s index finger into the buccal vestibule and gently retracting the cheek laterally, during which increased pliability and reduced fibrotic resistance were observed. These soft-tissue improvements corresponded clinically with the resolution of the patient’s previously restricted mouth opening and enhancement in his ability to masticate and retain food within the oral cavity. Additional treatments for newly developed lesions were provided throughout the follow-up period but are not detailed within this report.

The clinical team reported no adverse events, and the patient’s parents reported that the occlusal guard was well tolerated by the patient and showed notable clinical improvement over the 12-month follow-up period. They also mentioned that temperature monitoring via a wearable device and assessments with pediatrician-facilitated control of temperature fluctuations.

## 5. Discussion

The CIPA syndrome is the fourth type of HSAN caused by autosomal recessive mutations and polymorphisms in the NTRK1 gene, which encodes the receptor tyrosine kinase for nerve growth factor (NGF) [8]. Several labels have been used to characterize the HSAN, including congenital pure analgesia, congenital general pure analgesia, and congenital universal insensitivity to pain [7]. The principal criteria for CIPA syndrome are that the infant’s insensitivity to pain should affect the whole body, all other sensory modalities should be intact or slightly impaired, and tendon reflexes should be present [4]. The absence or dysfunction of small-diameter Aδ and C fibers in CIPA (HSAN IV) explains not only the hallmark analgesia but also impaired wound healing, defective thermoregulation, and the progressive oral fibrosis observed in this patient. These fibers mediate neurogenic inflammation, vasodilation, and neurotrophic support; therefore, their absence results in poor tissue repair, chronic ulceration, and increased susceptibility to infection [5,6].

From a pediatric and neurological standpoint, CIPA care should emphasize the prevention and early recognition of complications, rather than relying solely on symptom-driven presentations. Pediatric management priorities include education of caregivers about thermoregulation, proactive surveillance for infections or trauma, and co-management with neurology to screen for cognitive or autonomic comorbidities that affect safety and adherence to management plans [1]. Dental practitioners should be well versed in the clinical features of CIPA, including the potential for both intraoral and extraoral injuries, as well as relevant treatment considerations. When appropriate, dentists should consult with medical professionals to facilitate early diagnosis and management.

The current case diagnosis was primarily based on clinical assessment and supported by the Nociception Assessment Test and Minor’s Iodine–Starch Test [9,10]. The presence of consanguinity between the parents is consistent with the autosomal recessive inheritance pattern commonly observed in CIPA [11,12]. Given the absence of nociception (supported by the Nociception Assessment Test), anhidrosis (supported by the Minor’s iodine-starch test), recurrent painless fractures, oral self-injuries, and corroborative orthopedic and radiologic findings, the presumptive diagnosis of CIPA (HSAN IV) is established. However, the lack of genetic or histopathological confirmation remains a limitation of this case report.

Children with CIPA frequently experience multiple bone fractures that demonstrate delayed healing, often accompanied by hypertrophic callus formation and pseudoarthrosis. This phenomenon is attributable not only to congenital absence of pain perception but also to molecular alterations in nerve growth factor function, which disrupts the normal fracture-consolidation process [3]. The resulting atypical healing pattern is linked to the impaired development or proliferation of osteoprogenitor multipotent mesenchymal stromal cells, as well as aberrant differentiation of periosteal cells into fibroblasts. Deficiency of nociceptive fibers in both skin and skeletal tissues further affects bone metabolism, contributing to the high incidence of fractures and their substantial impact on patient function [13], as illustrated by the limb asymmetry and abnormal gait observed in the current case.

Edema of the lower limb and pelvis in this patient suggests a deep pelvic inflammatory process and possible hip dislocation, consistent with findings reported on bilateral hip MRI [10,14,15]. Previous studies have documented chronic arthritis and joint swelling in six of seven patients diagnosed with CIPA [11]. The absence of pain perception is believed to contribute to progressive hip joint deterioration and dislocation by compromising intrinsic joint-stabilizing mechanisms in affected individuals [16]. In the present case, pronounced edema of the left lower limb constituted a notable clinical finding.

Although early literature described high childhood mortality, particularly due to hyperthermia, more recent cohort data indicate that with comprehensive multidisciplinary care, many individuals with CIPA can survive into adolescence and adulthood [12]. Importantly, not all fatalities are attributable directly to the absence of pain; approximately half are associated with extreme hyperthermia, which may be unrecognized by patients and caregivers [11,17]. To mitigate this risk, we prescribed a wearable temperature-monitoring device for continuous detection of temperature fluctuations. To our knowledge, the use of such a device has not been documented in the dental management of a patient with CIPA. As reported by the patient’s parents, early detection of rising body temperature and timely intervention reduces the risk of temperature fluctuations. Given the high hyperthermia-related mortality reported by Klaitman et al. [12], this device has been assessed in RHA-CIPA monitoring and control, according to the patient’s parents.

The most prominent oral and para-oral characteristic of CIPA is the self-injury behaviors that result in lip, tongue, and cheek ulcerations, self-extraction of teeth, severe attrition and cervical abrasions, and erosion caused by gastroesophageal reflux due to faulty gastric motility, which causes feeding difficulties and repeated vomiting. Xerostomia results from repeated RHA-CIPA, which contributes to increased caries and oral infections (such as candidiasis and thrush). Insensitivity to thermal stimuli is a cause of mucosal burns, particularly the palate, from hot food or beverages [7,10,18]. The aforementioned clinical characteristics are consistent with the current case. The dental phenotype aims to ensure patient safety through the prevention and control of dental disease, as painless caries progression involving the dental pulp may lead to later space infection.

The patient’s parents requested full-mouth extraction and subsequent fabrication of complete dentures. Historically, similar management was reported in the 1960s, when the teeth of children with CIPA were extracted and replaced with full dentures [19]. However, such an approach is now considered excessively aggressive, as it may precipitate alveolar bone loss, negatively affect the child’s well-being, and disrupt normal jaw growth and permanent tooth eruption. Contemporary pediatric dentistry principles emphasize preservation of dentition whenever feasible to support normal orofacial development. A multidisciplinary, conservative treatment was planned instead, which showed improvement over a 12-month follow-up period.

The perioperative management of patients with CIPA presents notable challenges due to their increased risk of regurgitation, aspiration, hyperthermia, and bradycardia, all of which require meticulous monitoring and control throughout sedation or general anesthesia. The use of anticholinergic agents to treat bradycardia remains controversial because of their potential to exacerbate hyperthermia [20,21,22]. Consequently, multiple short, stress-free appointments were favored over a single visit under general anesthesia. A multidisciplinary behavior-management approach, including shortened appointments, the tell–show–do technique, and positive reinforcement, helped minimize patient fatigue, facilitate cooperation, and ultimately supported the decision to avoid general anesthesia. This management approach is consistent with prior observations by Shmueli et al. [23], who documented improved clinical outcomes within the context of a dedicated multidisciplinary care setting. It also accords with the updated oral and maxillofacial management recommendations for CIPA (HSAN IV) proposed by Yefet et al. [24], which underscore the importance of trauma prevention, coordinated interdisciplinary care, and rigorous perioperative monitoring. Management of the present case prioritized preventive strategies, brief low-stress clinical encounters, active parental participation in RHA-CIPA surveillance, and close collaboration with pediatric and orthopedic specialists.

There was a misconception that patients with CIPA do not require anesthetics because they do not perceive pain. In reality, they exhibit pronounced tactile hyperesthesia, which is thought to arise as a compensatory response to analgesia. Accordingly, a local anesthetic is necessary to prevent unexpected movements or defensive reactions during dental procedures [19,25]. Although pain and temperature perception are absent, proprioception and sensitivity to pressure, vibration, and touch are preserved [7], underscoring the need for intra-pulpal anesthesia for endodontic treatment and field-block local anesthesia for extractions in this patient. Local anesthesia was advocated for tooth extraction in the dental case report of CIPA in 1989 due to its vasoconstrictive benefits [19]. Subsequent reports have likewise emphasized the importance of local anesthesia when patients demonstrate discomfort during routine dental care [25].

Bone and cutaneous infections are the most commonly reported complications in individuals with CIPA, with Staphylococcus aureus identified as the most frequent pathogen [3,12]. Wound healing is often delayed and may be further compromised by self-injurious behaviors and cognitive impairment [26]. Accordingly, prophylactic systemic broad-spectrum antibiotic therapy was administered before the extraction procedure to minimize the risk of secondary infection [7]. Amoxicillin was prescribed for five days due to the chronic infection associated with the extracted tooth as a broad-spectrum systemic antimicrobial agent [12].

The use of mouthguards represents an important preventive strategy to protect children with CIPA from self-inflicted oral trauma until they acquire safer behavioral patterns. In this case, the patient exhibited restricted mouth opening caused by a thick fibrous band resulting from prolonged cheek biting, primarily attributed to nocturnal biting associated with parasomnia disorder. A hard acrylic maxillary protector was fabricated to control biting behavior and mitigate further self-injury, following the approach described by Littlewood and Hutton [27]. As observed during the 12-month review, this contributed to a reduction in lip and tongue biting, as well as improved cheek elasticity and tissue resilience.

### Limitations

This case was not part of a broader clinical investigation; rather, it is an observational single-case report that provides a detailed description of the oral phenotype and multidisciplinary management of a patient with CIPA. Several limitations must be acknowledged, including parental refusal of molecular testing and skin biopsy, the absence of preoperative quantitative mouth opening measures, and the lack of intraoral photography due to restricted opening and safety concerns. These limitations preclude inferential conclusions about the effectiveness of the intervention.

Quantitative pre-treatment measurements for maximum mouth opening were not recorded at baseline; therefore, postoperative comparisons could not be made. Restricted mouth opening and limited patient cooperation hindered the acquisition of high-quality intraoral photographs. High-quality intraoral photography requires sustained retraction, operator-controlled lighting, and stable cooperation; due to the patient’s limited and fatigue-sensitive opening, multiple attempts at intraoral photography provoked defensive movements and were considered unsafe. The intraoral scanner could be used because it requires less sustained retraction and can capture the dental arches in short passes. We therefore document intraoral findings using panoramic radiographs (Figure 6), digital scans, and clinical extraoral images (Figure 7).

The dental practitioners of the clinical team are not professionally qualified to manage RHA-CIPA; therefore, the patient’s medical care was supervised by a pediatrician, and clinical feedback was obtained from the parents. According to parental report, the patient demonstrated notable clinical management of the temperature fluctuations throughout the 12-month follow-up period.

The observations presented herein are descriptive in nature and pertain to a single clinical case; consequently, they should not be interpreted as evidence of therapeutic efficacy, causal relationships, or generalizability to the broader population of individuals with CIPA. Future research should incorporate emerging diagnostic and clinical tools, as well as novel management strategies, to advance the treatment of oral and para-oral manifestations associated with this condition.

This manuscript was prepared in accordance with the CARE reporting guideline [28], and adherence was verified using the CARE checklist [29]. The CARE checklist is provided in the Supplementary Materials as File S1, and the CARE Timeline Table is provided as Table S2.

## 6. Conclusions

This descriptive case report delineates the oral phenotype of a pediatric patient with a presumptive diagnosis of CIPA and outlines a multidisciplinary, conservative clinical management strategy. Although notable clinical improvements were documented during the 12-month follow-up, the findings remain inherently limited by the single-case design. Consequently, the effectiveness of any individual intervention cannot be generalized, and no causal relationships can be inferred.

The patient demonstrated adequate tolerance to a multidisciplinary treatment protocol characterized by brief, low-stress clinical encounters. The administration of local anesthesia appeared beneficial in mitigating discomfort associated with tactile hyperesthesia. Prophylactic broad-spectrum antibiotic therapy was implemented to decrease the likelihood of secondary infection, given the patient’s predisposition to self-inflicted oral trauma and impaired nociception. The incorporation of an intraoral scanner and the fabrication of a 3D-printed cast supported the delivery of minimally invasive dental procedures. Parental accounts regarding the utility of a temperature-monitoring device for identifying and managing hyperpyrexia, in coordination with pediatric care, remain subjective and do not constitute empirical evidence.

## Data Availability

The original contributions presented in this study are included in the article/Supplementary Material. Further inquiries can be directed to the corresponding author.

## Supplementary Materials

The following supporting information can be downloaded at: https://www.mdpi.com/article/10.3390/dj14010068/s1. File S1: CARE Checklist of information to include when writing a case report [29]; Table S2: CARE-Compliant Timeline Table for Dental Multidisciplinary Management; File S3: Clinical Report Ethical Approval.

## Abbreviations

The following abbreviations are used in this manuscript:

CIPA	Congenital Insensitivity to Pain and Anhidrosis.
RHA-CIPA	Recurrent Hyperpyrexia Associated with CIPA
NTRK1	Neurotrophic Tyrosine Kinase Receptor type 1.
HSAN	Hereditary Sensory and Autonomic Neuropathies.
NGF	Nerve Growth Factor
