# Supplementary material for: Multidisciplinary Approach for Dental Management of Congenital Insensitivity to Pain with Anhidrosis: Clinical Case Report with 12-Month Follow-Up
Source: Dent J (Basel). 2026 Jan 20;14(1):68. doi: 10.3390/dj14010068 (PMC12840396; doi:10.3390/dj14010068)
Supplement: Supplementary file 1 [file dentistry-14-00068-s001.zip › Table S1.pdf]

CARE-Compliant Timeline Table for Dental Multidisciplinary Management

Date / Interval	Clinical Events & Findings	Interventions	Outcomes / Notes
**Initial Presentation (Age 9)**	• Multiple carious teeth, missing teeth, poor oral hygiene.<br>• Limited mouth opening due to fibrous cheek bands.<br>• Self-inflicted oral injuries (lips, tongue, buccal mucosa).	• Multidisciplinary, conservative management plan formulated.<br>• Parents counseled; informed consent obtained.<br>• Wearable temperature-monitoring device prescribed.	• Full-mouth extraction request declined.<br>• Treatment plan accepted by parents.
**Visit 1 – Surgical Session**	• Chronically infected root of left mandibular first molar.	• Attempted extraction without anesthesia triggered discomfort → field-block anesthesia administered.<br>• Extraction performed; socket sutured.<br>• Amoxicillin (pre-op and 5 days post-op) to prevent infection.	• Uneventful healing anticipated.<br>• Reinforced oral hygiene and wound-care instructions.
**Visit 2 – Endodontic Session**	• Maxillary left first molar with symptomatic pathology.<br>• Limited mouth opening impacted accessibility.	• Initial attempt without anesthesia triggered discomfort → intrapulpal anesthesia given.<br>• Full endodontic treatment performed<br>• Immediate glass ionomer coronal restoration.	• Procedure completed successfully despite limited access.
**Visit 3 - Restorative Session**	• Multiple carious teeth.<br>• Sharp tooth edges contribute to soft-tissue trauma.	• Glass ionomer restorations placed.<br>• Sharp edges smoothed.<br>• Work performed without anesthesia, tolerated by the patient.	• Pain-free tolerance; reduced risk of mucosal laceration.<br>• Intraoral photographs not obtainable due to limited opening.

**Visit 4 - Orthodontic/Behavioral Management**	• Persistent maladaptive habits: nocturnal biting of the cheek, lip, tongue.<br>• Masticatory muscle tightness.	• Habit-reversal and habit-termination counseling.<br>• Daily masticatory muscle stretching exercises taught and supervised.	• Improved cooperation and progressive soft tissue adaptation expected.
**Occlusal Protector Fabrication Phase**	Severe restrictions in mouth opening prevented the placement of the impression tray.	• Intraoral scanner used for digital impression.<br>• 3D-printed maxillary cast created.<br>• Hard acrylic occlusal guard (3 mm) fabricated, no palatal coverage.	• Appliance delivered with hygiene and night-time-use instructions.<br>• Designed to reduce nocturnal biting and self-injury.
**3-Month Follow-Up**	• Healing extraction site.<br>• No complaints from restorations.<br>• Improved oral hygiene.	• Continued appliance use.<br>• Continued stretching exercises.	• Reduction in new trauma.<br>• Parents report effective temperature monitoring for detection of temperature fluctuations.
**6-Month Follow-Up**	• Good compliance with the guard.	• Routine monitoring.	• Reduction in lip and tongue biting.<br>• Healing of prior tongue ulcer.
**12-Month Follow-Up**	• improved cheek elasticity and soft-tissue resilience.<br>• Improved mouth opening, mastication, and bolus control.<br>• Healing tongue ulcers; single new self-inflicted facial ulcer.	• Continued use of occlusal guard.<br>• Reinforcement of oral hygiene and habit-con	• The clinical team reported no adverse events.<br>• Patient's parents reported that the occlusal guard was well-tolerated by the patient<br>• Parents reported that temperature monitoring via a wearable device facilitated early detection of temperature fluctuations.
