# Supplementary material for: Multidisciplinary Approach for Dental Management of Congenital Insensitivity to Pain with Anhidrosis: Clinical Case Report with 12-Month Follow-Up
Source: Dent J (Basel). 2026 Jan 20;14(1):68. doi: 10.3390/dj14010068 (PMC12840396; doi:10.3390/dj14010068)
Supplement: Supplementary file 1 [file dentistry-14-00068-s001.zip › File S1.pdf]

CARE case report guidelines

# CARE Checklist of information to include when writing a case report

(cc) BY-NC-ND

| Topic | Item | Checklist item description | Reported on Line |
|---|---|---|---|
| Title | 1 | The diagnosis or intervention of primary focus followed by the words "case report" | Page 1, lines 2-3 |
| Key Words | 2 | 2 to 5 key words that identify diagnoses or interventions in this case report, including "case report" | Page 1, lines 36-38 |
| Abstract (no references) | 3a | Introduction: What is unique about this case and what does it add to the scientific literature? | Page 1, lines 11-15 |
| | 3b | Main symptoms and/or important clinical findings | Page 1, lines 15-18 |
| | 3c | The main diagnoses, therapeutic interventions, and outcomes | Page 1, lines 19-30 |
| | 3d | Conclusion—What is the main "take-away" lesson(s) from this case? | Page 1, lines 31-35 |
| Introduction | 4 | One or two paragraphs summarizing why this case is unique (**may include references**) | Page 1-2, lines 40-71 |
| Patient Information | 5a | De-identified patient specific information. | Page 2, line 74 |
| | 5b | Primary concerns and symptoms of the patient. | Page 2, lines 75-80 |
| | 5c | Medical, family, and psycho-social history including relevant genetic information | Pages 2-4, lines 83-107 |
| | 5d | Relevant past interventions with outcomes | No past interventions |
| Clinical Findings | 6 | Describe significant physical examination (PE) and important clinical findings. | Pages 4-5, lines 109-134 |
| Timeline | 7 | Historical and current information from this episode of care organized as a timeline | Supplement B |
| Diagnostic Assessment | 8a | Diagnostic testing (such as PE, laboratory testing, imaging, surveys). | Page 5, lines 144-149 |
| | 8b | Diagnostic challenges (such as access to testing, financial, or cultural) | Financial challenges |
| | 8c | Diagnosis (including other diagnoses considered) | Page 5, lines 135-154 |
| | 8d | Prognosis (such as staging in oncology) where applicable | Not applicable |
| Therapeutic Intervention | 9a | Types of therapeutic intervention (such as pharmacologic, surgical, preventive, self-care) | Pages 6-8, lines 163-241 |
| | 9b | Administration of therapeutic intervention (such as dosage, strength, duration) | Page 6, lines 189-192 |
| | 9c | Changes in therapeutic intervention (with rationale) | Not applicable |
| Follow-up and Outcomes | 10a | Clinician and patient-assessed outcomes (if available) | Pages 8-9, lines 244-276 |
| | 10b | Important follow-up diagnostic and other test results | Pages 8-9, lines 250-273 |
| | 10c | Intervention adherence and tolerability (How was this assessed?) | Missed |
| | 10d | Adverse and unanticipated events | Page 9, lines 268-273 |
| Discussion | 11a | A scientific discussion of the strengths AND limitations associated with this case report | Pages 12, lines 387-409 |
| | 11b | Discussion of the relevant medical literature **with references**. | Pages 10-12, lines 279-386 |
| | 11c | The scientific rationale for any conclusions (including assessment of possible causes) | Page 13, line 414 |
| | 11d | The primary "take-away" lessons of this case report (without references) in a one paragraph conclusion | Page 13, lines 410-422 |
| Patient Perspective | 12 | The patient should share their perspective in one to two paragraphs on the treatment(s) they received | Page 13, lines 420-422-377 |
| Informed Consent | 13 | Did the patient give informed consent? Please provide if requested | Yes ☒ No ☐ |
