# Supplementary material for: Multidisciplinary Approach for Dental Management of Congenital Insensitivity to Pain with Anhidrosis: Clinical Case Report with 12-Month Follow-Up
Source: Dent J (Basel). 2026 Jan 20;14(1):68. doi: 10.3390/dj14010068 (PMC12840396; doi:10.3390/dj14010068)
Supplement: Supplementary file 1 [file dentistry-14-00068-s001.zip › File S2.pdf]

Clinical Report Ethical Approval

Our manuscript presents a case report describing a novel clinical observation in the context of a rare disease with unexpected reactions. It does not constitute a clinical trial, nor does it meet the criteria for research that would require ethics committee (IRB/REC) approval or trial registration because the report describes the management of the patient's disease as a regular treatment without any experimental or research interventions. The key justifications are outlined below:

The primary aim of our case report is to share a unique clinical observation encountered during routine medical care. We did not intend to conduct a formal research study or evaluate the effects of any intervention. No research questions, study hypotheses, or structured objectives were planned.

Our manuscript does not follow a research study design. There was no study protocol, no predefined outcomes, and no methodology intended to answer a research question or test a hypothesis. The report is purely observational, based on the clinical course of a single patient.

Unlike clinical trials, which are hypothesis-driven and often include control groups, randomization, and predefined metrics, our case report is a descriptive account. It does not involve sample size calculation, study arms or comparison groups, randomization or blinding, evaluation of materials, techniques, or procedures, use of outcome measures or scales, or statistical analysis.

The manuscript discusses an individual case, which lacks the scope and methodology of a clinical trial. Clinical trials typically involve multiple participants and are designed to produce generalizable results through systematic testing.

The interventions and decisions described followed standard clinical practice. No investigational treatment or experimental procedures were introduced outside of accepted medical care.

In conclusion, our case report differs fundamentally from a clinical trial in its purpose, design, methodology, and intent. It represents a descriptive account of an individual patient in routine care, without the features of interventional research. Therefore, it does not require ethics approval or registration, based on widely accepted ethical guidelines such as ICMJE, COPE, and national research standards. Since our manuscript is not a clinical trial, it does not require the CONSORT 2025 checklist for randomized controlled trials or the TREND checklist for non-randomized trials because the report does not have multiple participants. However, we obtained informed consent from the patient’s parents

for approval of our treatment planning and a written consent for publication of the manuscript. All necessary steps were taken to ensure confidentiality and ethical integrity.
